# Chemical ontologies: what are they, what are they for and what are the challenges

**DOI:** 10.1186/1758-2946-3-S1-O4

**Published:** 2011-04-19

**Authors:** Janna Hastings, Nico Adams, Marcus Ennis, Duncan Hull, Christoph Steinbeck

**Affiliations:** 1 Chemoinformatics and Metabolism team, European Bioinformatics Institute, Hinxton, UK

## 

Ontologies encode human knowledge in computationally accessible forms. They are designed to narrow the gap between the knowledge of human experts and the functionality available in computer systems, by expressing expert knowledge in a manner computers can manipulate and reason over. With the ever-growing deluge of data in modern scientific domains, researchers need intelligent tools able to filter out irrelevant and automatically organise relevant information into meaningful categories. The Chemoinformatics and Metabolism team at the EBI is developing chemical ontologies for structure-based chemical classification, role or bioactivity-based chemical classification, and chemical information entities such as descriptors and algorithms. Our ontologies provide collections of names and synonyms which are useful for text mining, stable identifiers which are essential to semantic integration of data, and a semantically rich encoding of many aspects of the chemical domain. But for such ontologies to be maximally useful for diverse users and interoperable with other ontologies in the scientific domain, similar-sounding things have to be disentangled in our language and our ontology. Recent work addresses the distinguishing of structures from chemicals [1], and of bioactivity from drug uses [2]. Ontologies are backed by logical formalisms such as the Web Ontology Language, OWL. One of the challenges of chemical ontology is representing complex chemical structures in the underlying formalism. Cyclic structures prove particularly challenging for logic-based representation. Recent research in our group investigated the inclusion of chemical graphs in OWL [3]. Integrating chemoinformatics tools with chemical ontology is the subject of ongoing research.
